# Spatial range, temporal span, and promiscuity of CLE-RLK signaling

**DOI:** 10.3389/fpls.2022.906087

**Published:** 2022-08-26

**Authors:** Madhumitha Narasimhan, Rüdiger Simon

**Affiliations:** ^1^Institute for Developmental Genetics, Heinrich-Heine University, Düsseldorf, Germany; ^2^Institute for Developmental Genetics and Cluster of Excellence in Plant Sciences, Heinrich-Heine University, Düsseldorf, Germany

**Keywords:** CLE, RLK, proliferation, differentiation, spatial range, non-developmental effects, promiscuity, co-evolution

## Abstract

CLAVATA3/EMBRYO SURROUNDING REGION-RELATED (CLE) signaling through receptor-like kinases (RLKs) regulates developmental transitions and responses to biotic and abiotic inputs by communicating the physiological state of cells and tissues. CLE peptides have varying signaling ranges, which can be defined as the distance between the source, i.e., the cells or tissue that secrete the peptide, and their destination, i.e., cells or tissue where the RLKs that bind the peptide and/or respond are expressed. Case-by-case analysis substantiates that CLE signaling is predominantly autocrine or paracrine, and rarely endocrine. Furthermore, upon CLE reception, the ensuing signaling responses extend from cellular to tissue, organ and whole organism level as the downstream signal gets amplified. CLE-RLK-mediated effects on tissue proliferation and differentiation, or on subsequent primordia and organ development have been widely studied. However, studying how CLE-RLK regulates different stages of proliferation and differentiation at cellular level can offer additional insights into these processes. Notably, CLE-RLK signaling also mediates diverse non-developmental effects, which are less often observed; however, this could be due to biased experimental approaches. In general, CLEs and RLKs, owing to the sequence or structural similarity, are prone to promiscuous interactions at least under experimental conditions in which they are studied. Importantly, there are regulatory mechanisms that suppress CLE-RLK cross-talk *in vivo,* thereby eliminating the pressure for co-evolving binding specificity. Alternatively, promiscuity in signaling may also offer evolutionary advantages and enable different CLEs to work in combination to activate or switch off different RLK signaling pathways.

## Introduction

Cell to cell communication in plants is co-ordinated by several mobile signals, such as peptides, hormones, RNAs, and proteins. The CLE family of peptides in plants is central for communication and mediation of a wide range of processes that are essential for development, and in response to biotic and abiotic stimuli. The CLE pre-propeptides with a size of 80–100 amino acids are first synthesized in the rough endoplasmic reticulum in an inactive form, and then proteolytically processed and have been, in some cases, shown to be glycosylated in the secretory pathway to form an active peptide of 12 or 13 amino acids, consisting of a highly conserved CLE domain as it reaches the apoplast. CLE peptides then bind to plasma membrane-localized RLKs on the CLE-secreting or adjacent cells, or are transported *via* the phloem to trigger intracellular signaling cascades in distant target tissues (Rojo et al., 2002; Gao and Guo, 2012; Notaguchi and Okamoto, 2015). Based on their function and sequence similarities, CLE peptides can be broadly classified into A-type (CLAVATA3 (CLV3) and CLV3-likes) that promote differentiation, or B-type (TRACHEARY ELEMENT DIFFERENTIATION INHIBITORY FACTOR (TDIF) and TDIF-likes) that promote proliferation when applied exogenously (Whitford et al., 2008). CLE peptides are often perceived by leucine-rich repeat (LRR)-RLKs, which contain an extracellular ligand-binding domain consisting of LRRs, a transmembrane domain, and an intracellular kinase domain (Poliushkevich et al., 2020). In this review, we will focus on CLE interaction with LRR-RLKs (hereafter referred to as CLE-RLK). CLE peptide perception may also require additional receptors for its perception and initiation of signaling cascade, such as the receptor-like protein CLAVATA 2 (CLV2), which contains an extracellular receptor domain, a transmembrane domain, and a small cytoplasmic tail, and the receptor-like pseudokinase CORYNE (CRN), which contains a transmembrane domain and a kinase domain. While CLE peptides almost exclusively bind and signal through members of the RLK subfamily XI, the co-receptors are from other subfamilies (Poliushkevich et al., 2020; Furumizu et al., 2021). RLKs can form homomers at the plasma membrane and interact with CLV2-CRN heteromers in the absence of the peptide ligand; notably, these preformed complexes cluster after CLE peptide binding (Bleckmann et al., 2010; Somssich et al., 2015; Hu et al., 2018). However, the precise function of the CLV2-CRN pseudo-receptor heteromeric complex is not yet known.

## CLE-RLK signaling and effects

CLE-RLK signaling is a spatially co-ordinated process and its effects are highly temporally correlated. The spatial dimension constitutes the cells/tissue layers that send out the CLE signal, and those that perceive these signals through RLKs and respond to them, i.e., the signaling range (Müller and Schier, 2011). CLE peptides can be secreted and perceived by the same cell or a group of cells (autocrine). Alternatively, the peptide molecules can diffuse to the nearby cells to signal (paracrine), or can travel long-distance to different organs (endocrine). In this section, we have done a case-by-case analysis of some imperative and recent studies pertaining to the origin and the destination of the CLE signal.

The events following CLE perception have a wide temporal range. For example, activation of transcription factors (TFs) and subsequent induction of responsive genes happens in minutes, control of cell division occurs after hours, establishment of primordium occur in a day, formation of new organs require several days. Thus, the ensuing cell to organ level effects vary as the downstream signal gets amplified and spreads through intercellular communication (Plieth, 2010; Van Norman et al., 2011). This section highlights the temporal dimension of CLE-RLK signaling and their multiple effects—from molecular or cellular level (considered as short-term effect) to tissue, organ, or organism level (considered as long-term effect)—Table 1.

**Table 1:** Summary of CLE-RLK signaling.

CLE peptide	RLK and/or other receptors involved	Origin of the peptide	Destination of the peptide/location of RLK	Short-term (molecular, cellular) and long-term (tissue, organ, organism) effects
			Signaling range	
**In the apical meristem of *Marchantia***				
**MpCLE2**	Signals through MpCLV1	The apical notch but outside the central region that hosts central subapical cells	The meristem with the apical and subapical cells where *MpCLV1* is expressed	Short-term: Inhibits differentiation of the subapical cells **Long-term: Mediates accumulation of subapical cells and enables their subsequent differentiation and dichotomous branching**
			**Paracrine signaling**	
**MpCLE1**	Signals through MpTDR	The ventral part around the apical cell	Dorsal part where *MpTDR* is expressed	Short-term: Inhibits proliferation of the apical meristem **Long-term: Regulates expansion of the thallus and proper formation of gametangiophores and gemma cups**
			**Both auto- and paracrine signaling**	
**During *Physcomitrium* gametophore formation**				
**PpCLEs 1, -2, and -7**	(PpCLE1 to -7) Signal through PpRPK2 and PpCLV1a and -b	Different regions in the gametophores	Gametophore	Short-term: Initiation of formative division, maintenance of CD orientation and specification of cell fate **Long-term: 1. Mediates formation of properly sized mature gametophores 2. Inhibits proliferative divisions in gametophore, thus maintaining gametophore and leaf size**
**PpCLE6**		Protonemal filament	Likely gametophore	
**PpCLE 3, -4, and -5**		Not characterized			Likely gametophore
**In the SAM of *Arabidopsis***					
**CLE40**	Signals through (and likely binds) BAM1	PZ of IFM and SAM	PZ where *BAM1* is expressed	Short-term: 1. Induces *WUS* in the OC 2. Promotes proliferation and suppresses differentiation of stem cells **Long-term: 1. Promotes SAM growth 2. Proper formation of floral organs**
			**Likely autocrine signaling**	
**CLV3**	Binds and signals through CLV1; signals through RPK2, CLV2/CRN	CZ of IFM and SAM	OC where it binds and signals through CLV1	Short-term: 1. Represses *WUS* and its expansion into CZ 2. Suppresses proliferation of stem cells and enables their differentiation **Long-term: 1. Inhibits SAM growth 2. Regulates proper formation of floral organs**
			**Paracrine signaling**	
**In the root apical meristem of *Arabidopsis***				
**CLE45**	Binds BAM3; signals through CLV2/CRN, RPK2	PSE and SPC	1. PSE and SPC where it binds and signals through BAM3	Short-term: 1. Inhibits periclinal, formative division of SPC into proto- and metaphloem cells 2. Inhibits the acquisition of morphological changes during PSE differentiation **Long-term: 1. Regulates PSE cell file formation 2. Regulates PRM development**
			**Autocrine signaling**	
			2. Likely CC and PPP where *RPK2* is expressed	Short-term: Inhibits CC and PPP differentiation into PSE **Long-term: Maintains a reservoir of phloem cells with plastic identity for future needs**
			**Likely paracrine signaling**	
**CLE25**	Signals through CIK and CLV2	In root: SPCs and its lineage; In stem: sieve elements	Not characterized	Short-term: Regulates periclinal, formative division of SPC into proto- and metaphloem cells **Long-term: Regulates PRM development, phloem transport and starch immobilization**
**In the vascular meristem**				
**CLE41**	Signals through PXY	Phloem	Cambium where *PXY* is expressed	Short-term: 1. Triggers proliferation of procambium/cambium by upregulating *WOX4* 2. Inhibits its differentiation into xylem 3. Controls orientation of procambial cell division **Long-term: 1. Organized vascular patterning 2. Maintenance of stele size and radial growth of the vascular system**
			**Paracrine signaling**	
**PttCLE41**	Likely signals through PttPXY	Phloem	Likely vascular cambium where *PttPXY* is expressed	Short-term: Likely induces proliferation of cambial cells, which then differentiate into xylem cells **Long-term: 1. Maintains overall secondary vascular growth and stem diameter 2. Regulates the internodal length and height of the plant**
			**Likely paracrine signaling**	
**PtrCLE20**	Likely signals through PtrCLV2	Xylem	Vascular cambium	Short-term: Likely suppresses cambial cell proliferation leading to a decreased rate of xylem differentiation **Long-term: Maintains overall secondary vascular growth and stem diameter 2. Regulates the internodal length and height of the plant**
			**Paracrine signaling**	
**CLE9**	Binds BAM1; signals through BAM2 and -3	Xylem precursors, particularly of protoxylem cell file positions	Likely xylem precursors of protoxylem cell file positions, where it binds and signals through BAM1, although BAM1 is broadly expressed in vascular and pericycle cells	Short-term: Prevents peri- and anticlinal divisions of xylem precursors that increases xylem and procambial cell number **Long-term: 1. Regulates the number of xylem and procambium cell files 2. Regulates the overall plant growth**
			**Likely autocrine signaling**	
**In stomatal lineage development**				
**CLE9**	Binds HSL1-SERK1, the RLK-coreceptor complex	MMC, meristemoids and GCs	MMC and meristemoids, where it binds and signals through HSL1	Short-term: 1. Destabilizes SPCH to prevent MMC from acquiring its identity 2. Prevents its further asymmetric divisions **Long-term: Regulates the density of GCs and PCs in leaves**
			**Likely autocrine signaling**	
**In root nodulation**				
**MtCLE12 and -13**	Signals through MtSUNN, MtCRN	Nodule primordium in root	Likely shoot where *MtSUNN* is expressed	Short-term: MtCLE13 suppresses the proliferative divisions likely right after the initial cell divisions of the cortex and pericycle **Long-term: 1. Both peptides inhibit nodule primordium development 2. Decrease the nodule numbers, thus establishing N homeostasis**
			**Likely endocrine signaling**	
**LjCLE-RS1 and -2**	Binds LjHAR1; signals through LjKLV and LjCLV2	Nodule primordium in root	Shoot, likely in leaf phloem, where *LjHAR1* is expressed	Short-term: LjCLE-RS1 and −2 peptides negatively regulate continuous cortical cell divisions after a few rounds of initial divisions **Long-term: 1. Both peptides inhibit nodule primordium development 2. Decrease the nodule numbers, thus establishing N homeostasis**
			**Endocrine signaling**	
**In nematode infection**				
**HsCLEB**	Signals through TDR, CLV1, RPK2, CLV2/CRN	Nematode esophageal gland	Likely procambial cells in the root and/or the syncytial cells expressing TDR	Short-term: Induces procambial proliferative divisions **Long-term: Induces syncytia formation and increases rate of infection**
**In organ primordium and organ development**				
**CLE26**	Can bind and possibly signals through BAM1 and -2	Phloem pole of the stele in basal meristem	Not characterized	Short-term: Affects the PIN1 protein level in the root **Long-term: 1. Alters the auxin distribution in roots 2. Regulates PR length and LR density**
**CLE3**	Signals through CLV1	Pericycle cells in PR and LR	Likely companion cells where *CLV1* is expressed	Short-term: Not characterized **Long-term: 1. Inhibits LR emergence 2. Prevents root expansion in low N conditions**
			**Likely paracrine signaling**	
**CLE5**	Not characterized	Bases of young rosette leaves, of cauline leaves and of cotyledons of mature embryo; at both the adaxial and abaxial domains of vegetative shoot apex in developing rosette leaves	Not characterized	Short-term: Not characterized **Long-term: Regulates leaf width and symmetry**
**CLE6**	Not characterized	Bases of young rosette leaves and floral organs; only at the adaxial domain of vegetative shoot apex in developing rosette leaves	Not characterized	Short-term: Not characterized **Long-term: Regulates leaf width, symmetry and curvature**
**Not characterized**	CLV2/CRN	Not characterized	Not characterized	Short-term: 1. Upregulates auxin synthesis genes in the IFM cells 2. Maintains PIN1 protein levels in the IFM cells **Long-term: 1. Maintains overall auxin signaling in the IFM 2. Mediates flower primordia outgrowth and complete flower formation**
**CLV3 and other CLEs**	Signal through BAM1, -2, and -3	Not characterized	Not characterized	Short-term: Not characterized **Long-term: 1. Mediate flower primordia outgrowth and complete flower formation**
**In regulation of non-developmental responses**				
**CLE25**	Signals through BAM1 and -3	Vascular procambium of, possibly, the root	Leaf where *BAM1* and -2 are expressed	Short-term: 1. Promotes ABA synthesis 2. Enables stomatal closure **Long-term: Reduces water loss and ensures overall survival of the plant during water deficiency**
			**Endocrine signaling**	
**CLE9**	Not characterized	Stomatal GCs	Likely the stomatal GCs	Short-term: 1. Activates *MPK3,−6* 2. Enables stomatal closure by signaling through effectors, such as, ABA, NO, H_2_O_2_ **Long-term: Prevents excessive water loss, thus conferring resistance to drought stress**
			**Likely autocrine signaling**	
**MtCLE53**	Signals through MtSUNN	Vascular tissue with increased expression near colonization sites	Not characterized	(MtCLE53/-33) Short-term: 1. Upregulates *MtSUNN* 2. Suppresses the expression of strigolactone biosynthetic genes **(MtCLE53/-33) Long-term: Suppresses excessive AM fungal colonization thus attaining Pi homeostasis**
**MtCLE33**	Signals through MtSUNN	Vascular tissue with strong expression in pericycle and xylem parenchyma (no change due to colonization)	Not characterized	
**RiCLE1**	Not characterized	Fungi colonizing the root	Likely the epidermal and cortical cells	Short-term: Not characterized **Long-term: 1. Modulates root architecture by promoting PR and LR branching 2. Promotes the entry and spread of the fungi**
**CLE45**	Binds SKM1 and signals through SKM1 and -2	Stigma of the pistil at 22°C and expanded to the transmitting tract where pollen elongates at 30°C	Pollen where it binds SKM1	Short-term: Retains mitochondrial dehydrogenase activity at high temperature, thus prolonging pollen viability 2. Sustains pollen performance and increases the chances of pollen tubes reaching the ovules **Long-term: Ensures stable seed production**
			**Paracrine signaling**	

The list of CLE peptides and RLKs and other receptors they bind and/or signal through, their signaling range, the origin of the peptide and its destination where it exerts its effect, the short- and long-term effects covering the molecular, cellular, tissue, organ, and organism levels. ABA, abscisic acid; AM, arbuscular mycorrhiza; BAM, BARELY ANY MERISTEM; CC, companion cells; CD, cell division; CIK, CLAVATA3 INSENSITIVE RECEPTOR KINASES; CLE, CLAVATA3/EMBRYO SURROUNDING REGION-RELATED; CRN, CORYNE; CLV, CLAVATA; CZ, central zone; GC, guard cell; HSL, HAESA-LIKE 1; IFM, inflorescence meristem; LR, lateral root; MMC, meristemoid mother cell; MPK, MITOGEN-ACTIVATED PROTEIN KINASE; OC, organizing center; PC, pavement cell; PIN, PIN-FORMED; PPP, phloem pole pericycle; PRM, proximal root meristem; PSE, protophloem sieve element; PXY, PHLOEM INTERCALATED WITH XYLEM; PZ, peripheral zone; RLK, receptor-like kinase; RPK2, RECEPTOR-LIKE PROTEIN KINASE 2; SAM, shoot apical meristem; SKM, STERILITY-REGULATING KINASE MEMBER; SPC, sieve element precursor cell; SPCH, SPEECHLESS; SERK, SOMATIC EMBRYOGENESIS RECEPTOR KINASE; SUNN, SUPER NUMERIC NODULES; TDR, TDIF RECEPTOR; WOX, WUSCHEL-related HOMEOBOX 4; WUS, WUSCHEL. The name of the CLE peptide, its signaling range and the long term effects it mediates are described in bold letters.

### Regulation of proliferation and differentiation

Proliferation is the increase in the number of cells with the same identity as a result of continuous symmetric cell divisions, whereas differentiation involves symmetric or asymmetric formative divisions resulting in cells with different identities. CLE-RLK signaling regulates proliferation and differentiation of meristems, the region that embodies pluripotent stem cells, which continuously supply proliferating cells in the plant body. Plant and non-plant CLE peptides also regulate primordium formation in the root during bacterial symbiosis and nematode infections. Such CLE-RLK mediated regulation of proliferation and differentiation have been reported largely at the organ and tissue levels (Fletcher, 2020; Whitewoods, 2021; Willoughby and Nimchuk, 2021). However, the regulatory events occurring at the cellular level in controlling cell division and differentiation have not been understood well.

**Figure 1:**
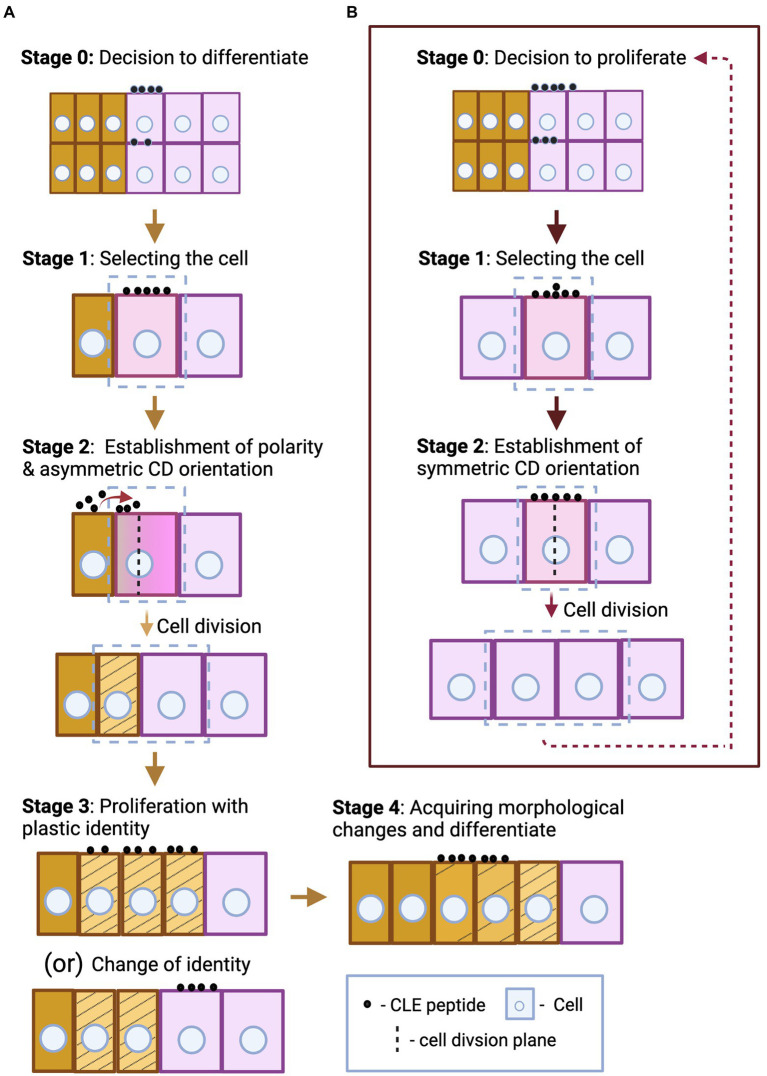
Schematic representation of some key aspects of different stages of asymmetric stem cell division and differentiation **(A)**, and symmetric cell division during proliferation **(B)**. **(A)** In stage 0, the CLE peptides that originate from the same or the surrounding cells send the signal to differentiate. Following the signal, the stem cell undergoes differentiation, and this process can be divided into four basic stages. First, the cell to undergo asymmetric formative division is selected (marked by a box)—stage 1. Based on the positional cues offered by neighboring cells through secreted CLE peptides (their origin and direction of diffusion are indicated by red arrow), the cell polarity and the cell division orientation are established. The cell divides producing daughter cells of unequal sizes with distinct identity (represented by striped yellow and pink cells)—stage 2. When the freshly divided stem cells are endowed with plastic identity (represented by stripes), they proliferate with the same plastic identity, or a particular cell (striped yellow) switches its identity to that of the surrounding cell (pink), under certain circumstances—stage 3. The cells further differentiate and reach its destination identity (unstriped yellow) by acquiring several morphological changes over time—stage 4. Stages 3 and 4 may also be regulated by CLE peptides, but the origin and directionality of the CLE peptides regulating stages 0, 3, and 4 are not defined in this figure as these can be case specific. **(B)** After the decision-making CLE peptides signal to proliferate—stage 0, the stem cells undergo proliferation, and this process comprises of two basic stages. First, the cell to undergo symmetric division is selected—stage 1. Then a symmetric cell division plane is set and the cell divides to form identical daughter cells—stage 2. The stem cells proliferate by continuous repetition of the two cell division stages. CLE peptides can induce or inhibit any of these stages of differentiation and proliferation processes.

**Figure 2:**
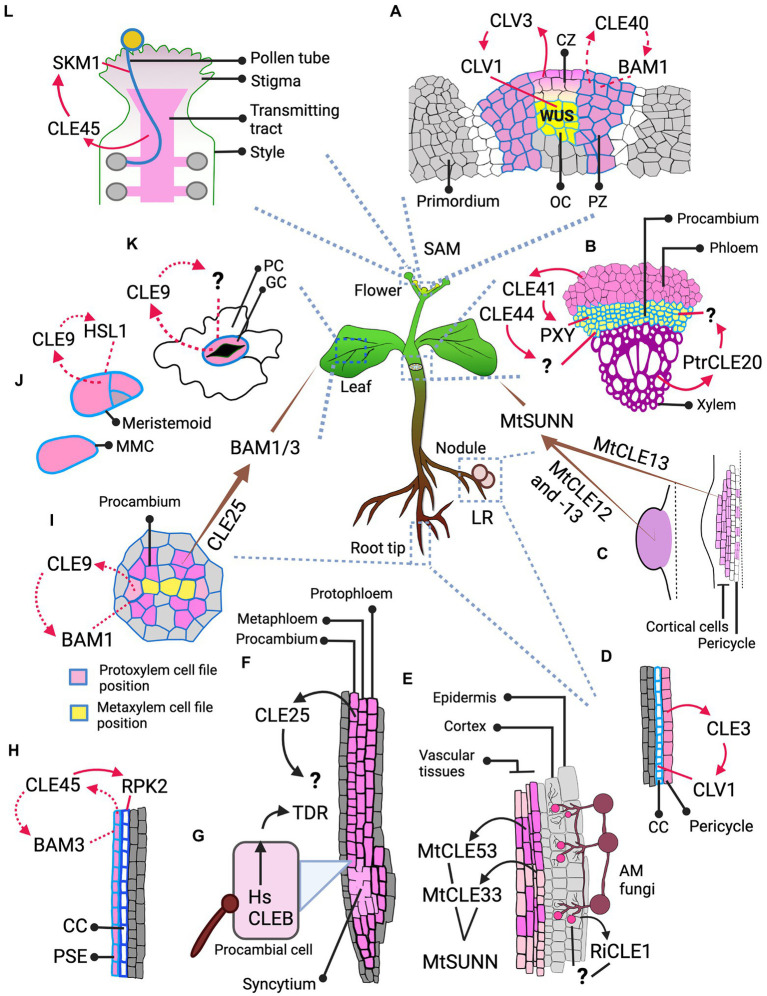
Spatial dimension of CLE signaling. The figure illustrates the cell or tissue of origin of the CLE peptide and its destination, and the RLK it binds to at the destination, thus defining whether the signaling type is autocrine, paracrine or endocrine. **(A)** Signaling in IFM: CLV3, as a paracrine signal from CZ, signals through CLV1 at the OC; CLE40, likely as an autocrine signal, signals through BAM1 at the PZ. **(B)** Paracrine signaling in vascular development: *Arabidopsis* CLE41 and CLE44 move from phloem to signal in procambium. CLE41 signals through PXY but the RLK through which CLE44 signals is unknown. In *Populus*, PtrCLE20 from xylem signals through an unknown RLK in procambium. **(C)** AON signaling from nodules: MtCLE13 from the cortical and pericycle cells of the developing nodule primordium (on the right), and both MtCLE12, -13 from young, round nodule (on the left) likely act as an endocrine signal and signal through MtSUNN in the shoot. **(D)** Signaling in LR: CLE3 from pericycle likely acts as a paracrine signal and moves to phloem CC to signal through CLV1. **(E)** Signaling during AM fungal symbiosis: *Rhizophagus irregularis* secretes RiCLE1 likely into the epidermal and cortical layers of the *Medicago* root, but the RLK that receives the CLE signal is unknown. *MtCLE53* and -*33* are expressed in the vascular tissues, while *MtCLE53* is upregulated close to the fungal infection sites. These CLEs signal through MtSUNN the localization of which is unknown. **(F,H)** Signaling during root protophloem development: **(F)** CLE25 is expressed in SPC lineage—protophloem, metaphloem, and procambium, but where and through which RLK it signals remains unknown. **(H)** CLE45, expressed in PSE, mediates an autocrine signaling regulation through BAM3 and likely a paracrine signaling regulation in CC through RPK2. **(G)** Signaling by nematode: HsCLEB, secreted by nematodes into the procambial cells, signals through TDR but it is not clear where it is expressed. **(I)** Autocrine signal in vascular development: CLE9, expressed in xylem precursors, signals through BAM1 expressed in vascular cells including xylem precursors. **(I–K)** Signaling in leaf: **(I)** CLE25, expressed in vascular procambium of root, acts as an endocrine signal and signals through BAM1/3 expressed in leaves for stomatal closure. **(J)** CLE9 expressed in MMCs and meristemoid cells binds HSL1 expressed in the same cells and likely mediates an autocrine signaling regulation during stomatal development. **(K)** CLE9 likely mediates an autocrine signal through uncharacterized RLK in stomatal GCs for stomatal closure. **(L)** Paracrine signaling in pollen. CLE45 that expands into the transmitting tract signals through SKM1 in pollen. Various shades of pink mark the cells expressing different CLEs. Cell outlines with varying shades of blue represents different RLKs. The arrows indicate the signaling type. The brown tapering arrow—endocrine signaling; red solid arrow—paracrine signaling; red dotted arrow—autocrine signaling; black arrows—unknown signaling type due to lack of data on the RLK and/or its localization. Abbreviated cells and tissues: CZ, central zone; CC, companion cell; GC, guard cell; LR, lateral root; MMC, meristemoid mother cell; PC, pavement cell; PSE, protophloem sieve element; PZ, peripheral zone.

There are several stages that lead to cell division and differentiation or proliferation at cellular level. When the need for proliferation and differentiation is evaluated, CLE peptides, in addition to other signaling factors, can be secreted to signal the decision. Then the stem cell that will undergo formative division (stage 1—Figure 1A) receives the signal. Formative divisions, which can be asymmetric or symmetric, give rise to daughter cells of different identities. During asymmetric divisions, external signals, such as CLE peptides from the surrounding cells, together with the differentially inherited and intrinsic proteins and microRNAs initially polarize the cell. Then the orientation of cell division sets and the cell divides forming daughter cells with two distinct identities (stage 2—Figure 1A; De Veylder et al., 2007; De Smet and Beeckman, 2011; Kajala et al., 2014; Xu et al., 2021). The freshly divided stem cells are initially endowed with plastic identity with which they proliferate. These cells, through cues from CLE peptides based on the needs of the surrounding cells, can remain uncommitted to their identity (stage 3—Figure 1A). But over time, the cells commit to their identity through several morphological changes and differentiate, which can also be regulated by CLE (stage 4—Figure 1A). However, fully differentiated plant cells are capable of de−/redifferentiation (Gilbert, 2000; De Smet and Beeckman, 2011; Furuta et al., 2014; Gujas et al., 2020; Seo et al., 2020). A similar set of events occurs during the proliferation process of meristematic stem cells or the cells forming the primordium (Figure 1B; De Veylder et al., 2007; Desvoyes et al., 2021). In addition to CLE peptides, several factors like hormones and TFs have been shown to regulate proliferation and differentiation at various stages (De Smet and Beeckman, 2011; Xu et al., 2021). This section highlights the influence of CLE-RLK signaling on stages of cell division and differentiation at the cellular level, in addition to the eventual tissue, organ and organism level effects (Figure 2; Table 1).

#### In the apical meristem of *Marchantia*

The genome of the liverwort *Marchantia polymorpha* encodes two CLE peptides: (1) *MpCLE1*, a TDIF-like peptide and (2) *MpCLE2*, a CLV3-like peptide, and two RLKs: *MpTDIF RECEPTOR* (*TDR*) and *MpCLAVATA 1* (*CLV1*). The apical meristem, hosted by the apical notch, is located at the growing tip of the gametophyte body (thallus). The apical notch, apart from being responsible for the radial expansion of the thallus, also produces gametangiophores, which are the reproductive structures developed from the thalli (Shimamura, 2016; Hirakawa et al., 2019). Hirakawa et al. (2019) showed that *Mpcle1* mutant exhibited thickening of the thallus and overgrowth of gametangiophores, whereas overexpression of *MpCLE1* resulted in small convoluted and distorted thalli. *MpCLE1*, expressed in the ventral part around the apical cell likely diffuses to the dorsal part where *MpTDR* is expressed to signal for suppression of proliferation (paracrine signaling; Hirakawa et al., 2019).

*MpCLE2* inhibits differentiation of the dorsal and ventral derivatives at the apical meristem. The apical notch laterally expanded upon *MpCLE2* overexpression as undifferentiated anticlinally divided subapical cells over-accumulated. This resulted in multichotomous branching, whereas subapical cells, after reaching threshold cell numbers, immediately divide periclinally to differentiate and undergo dichotomous branching. *MpCLE2* is normally expressed outside of the central subapical stem cell region and signals *via* MpCLV1, which is expressed in the central region. MpCLE2 likely moves to the central region to elicit inhibition of differentiation (paracrine signaling; Hirakawa et al., 2020).

To summarize, MpCLE1 and MpCLE2 signal through MpTDR and MpCLV1 to inhibit proliferation and differentiation processes, respectively. However, it is not clear at which stage of cell division from stage 0 to 2 they act.

#### During *Physcomitrium* gametophore formation

CLE peptides regulate both differentiation and proliferation in moss. Whitewoods et al. (2018) showed that multiple CLE-like peptide mutants and CLV1-like receptor mutants exhibited defects in 3D development during gametophore formation. Fewer mutant gametophores were formed, and they had a significantly reduced height. Gametophore initiation was inhibited due to defects in orientation of cell division already at the 2-cell and 4-cell stages during which cell identity is defined. Moreover, the mutants formed a callus-like mass at the gametophore base, indicating defective proliferation and cell identity regulation. Furthermore, external application of the CLE peptides resulted in stunted gametophore development to which the RLK mutant, *Ppreceptor-like protein kinase 2* (*Pprpk2*) was resistant, implying that PpCLEs regulate proliferation through PpRPK2 (Whitewoods et al., 2018). This indicates that components and functions of CLE signaling networks regulating both proliferation and differentiation balance are conserved across clades during land plant evolution. Therefore, additionally understanding at what stages each of the PpCLE peptides operate to influence differentiation and proliferation processes will inform us of the common courses of action that complex land plants could have evolved.

#### In the shoot apical meristem of *Arabidopsis*

In the *Arabidopsis* inflorescence meristem (IFM), two key CLE peptides, CLV3 and CLE40, are expressed in complementary regions: *CLV3* is expressed in the central zone (CZ), where stem cells proliferate at a very slow rate. Stem cell daughters can shift to the peripheral zone (PZ) where the cells proliferate at a higher rate and start acquiring organ identities (flower meristem or leaf primordium; Fletcher et al., 1999; DeYoung et al., 2006; Deyoung and Clark, 2008; Schlegel et al., 2021). In the PZ, the cells express the CLE40 peptide. The homedomain TF WUSCHEL (WUS) is expressed in the organizing center (OC) and moves to the CZ to promote stem cell proliferation and *CLV3* expression (Mayer et al., 1998). CLE40 from the PZ promotes *WUS* expression in the OC by signaling through the RLK BARELY ANY MERISTEM1 (BAM1) in the PZ (likely autocrine signaling), thus indirectly promoting stem cell proliferation at the CZ (Deyoung and Clark, 2008; Schlegel et al., 2021). CLV3 diffuses from the CZ to the OC where it binds to CLV1 and signals to repress *WUS* (paracrine signaling; Figure 2A), limiting the WUS activity in the CZ, thereby restricting stem cell proliferation to decrease the IFM size (Clark et al., 1995, 1997; Fletcher et al., 1999; Brand et al., 2000; Schoof et al., 2000; Rojo et al., 2002). Correspondingly, *cle40* mutant exhibited smaller SAM and *clv3* mutant had enlarged SAM with extra floral organs (Clark et al., 1995; Schlegel et al., 2021). CLV2/CRN, RPK2 and a family of co-receptors, CLAVATA3 INSENSITIVE RECEPTOR KINASES1-4 (CIKs) are also involved in CLV3 signaling (Kayes and Clark, 1998; Brand et al., 2000; Muller et al., 2008; Kinoshita et al., 2010; Shinohara and Matsubayashi, 2015; Nimchuk, 2017; Hu et al., 2018). The number of proliferating cells in the CZ, and by extension the amount of CLV3 they secrete continuously determine the amount of WUS being expressed, which in turn promotes stem cell proliferation. Thus, the CLV3 signal coming from stem cells determines their proliferation rate. Similarly, CLE40 secreted by the differentiating cells also control *WUS* expression, thus determining the proliferation rate of stem cells that will eventually differentiate. Together, CLV3 and CLE40 signaling can convey the current proliferation and differentiation status of CZ and PZ, respectively, thereby influencing decisions to proliferate or differentiate further (stage 0—Figure 1).

Furthermore, WUS promotes *CLV3* expression and suppresses *CLE40* expression (Yadav et al., 2011; Schlegel et al., 2021). These two interconnected negative feedback loops equilibrate the levels of WUS, CLE40 and CLV3 in the SAM, in order to balance the rates of proliferation and differentiation.

#### In the root apical meristem

In *Arabidopsis* roots, *CLE40* is expressed by the stele cells in the differentiation zone and mediates proliferation of the proximal root meristem (PRM—the root meristem part that extends from the quiescent center toward shoot, as opposed to the distal root meristem, DRM, that extends toward the root tip; Hobe et al., 2003; Stahl et al., 2009). *cle40* loss-of-function mutants exhibit a prematurely differentiated and therefore shorter proximal meristem. However, external application or overexpression of *CLE40* and, notably, of all the A-type peptides resulted in highly differentiated PRM, which depends on CRN-CLV2 (Fiers et al., 2005; Ito et al., 2006). In contrast, external application of *Arabidopsis* or poplar TDIFs induced proliferation and elongation of the PRM (Liu et al., 2016; Yang et al., 2020). Yang et al. (2020) further showed that TDIFs promote proximal and DRM size by regulating PIN-FORMED (PIN) mediated polar auxin transport. Thus, exogenous application of A-type or B-type peptide triggers a very generalized response in regulating differentiation and proliferation of the entire meristem, but how they regulate these processes at a cellular level is not yet understood.

*CLE45* is expressed in the sieve element precursor cell (SPC) and its descendant cells in the PRM, and it has been shown to mediate both auto and paracrine regulatory signaling (Figure 2H). SPC undergoes periclinal division to form proto- and metaphloem cells. Protophloem cells then proliferate with a “plastic” protophloem sieve element (PSE) identity (in the meristematic zone) and these cells eventually undergo subsequent differentiation and commitment to PSE cell file identity (in the transition and differentiation zones). The RLK BAM3 is also expressed in SPC and its descendant cells, binds CLE45 (Figure 2H) and inhibits differentiation of the PSE cell file in diverse ways. At the cellular level, CLE45 suppresses periclinal division of SPC and the eventual formation of the PSE cell file. Additionally, CLE45 inhibits cells from committing to PSE identity in the differentiation zone by preventing them from acquiring morphological changes like cell wall thickening (Depuydt et al., 2013; Rodriguez-Villalon et al., 2014; Gujas et al., 2020). Furthermore, at tissue level, CLE45 signaling inhibits PRM development and elongation; however, this might be an indirect consequence of the lack of a PSE cell file (Depuydt et al., 2013; Hazak et al., 2017). CLV2 and CRN are essential in mediating CLE45 signaling (Fiers et al., 2005; Hazak et al., 2017), and interestingly, CRN promotes BAM3 localization at the plasma membrane (Hazak et al., 2017). Another plasma membrane-localized protein, OCTOPUS (OPS), sequesters CRN thus interfering with CLE45 signaling. Higher OPS activity or protein level antagonizes the CLE45 signaling effect. Moreover, *ops* mutants have increased BAM3 level and also frequently fail to form PSE (Hazak et al., 2017; Breda et al., 2019). This illustrates that the interplay between OPS and CLE45 signaling components retains a balance in PSE identity commitment in the phloem.

In addition to CLE45 inhibiting SPC descendants from acquiring PSE identity through autocrine signaling, CLE45 also inhibits the companion cells (CC) and phloem pole pericycle (PPP) cells from differentiating into PSE likely through paracrine signaling. Cell files surrounding PSE have the ability to reprogram and acquire PSE identity. CLE45 from PSE cell file signals to the neighboring CC and PPP cell files expressing RPK2 (Figure 2H) to prevent these cells from switching their identity to PSE, thus retaining a pool of plastic phloem cells (Gujas et al., 2020). During PSE formation, CLE45-BAM3 signaling controls different stages of cellular differentiation processes. It can inhibit the switch of the plastic CC and PPP identity into PSE, which occurs at stage 3 of cellular differentiation, or it can inhibit the PSE cells from acquiring morphological changes, which is stage 4 of cellular differentiation (Figure 1A). It also can suppress SPC formative division—at some point from stages 0 to 2—of cell differentiation process.

Similar to CLE45, CLE25 also affects PSE formation and consequently PRM development. *CLE25* is expressed in sieve elements of roots (Figure 2F) and stems and the *cle25* mutant exhibited a mild delay in PSE development. However, the mutant version CLE25_G6T_ caused more pronounced defective downstream signaling (Ren et al., 2019). Plants expressing CLE25_G6T_ sustained suppression of periclinal division of SPC into proto- and metaphloem (stage 0, 1, or 2). This further led to defects in PSE differentiation during early seedling development. However, at later stages, these earlier PSE defects resulted in significant accumulation of starch in leaves due to compromised starch remobilization caused by defective phloem transport (Ren et al., 2019).

#### In vascular development

The procambial and cambial cells constitute the vascular meristems. During primary growth, the plant mainly develops along the apical-basal axis. Once primary growth stops, cambium differentiates from procambium and actively proliferates and differentiates into secondary xylem and phloem, thus causing radial thickening of roots and stems, which occurs massively in woody plants (Miyashima et al., 2013; Růžička et al., 2015).

In *Arabidopsis*, the TDIF peptides (CLE41 and -44) have been shown to diffuse from phloem to procambium and promote proliferation of procambial/cambial cells by inducing *WUSCHEL-related HOMEOBOX4* (*WOX4*) expression; in parallel, they also inhibit differentiation of cambial cells into xylem (Hirakawa et al., 2008, 2010; Kondo et al., 2014). Similarly, in poplar trees, PttCLE41 peptide produced in the phloem also likely moves to cambium to induce cambial cell proliferation. Cambial cells express the RLK *PttPHLOEM INTERCALATED WITH XYLEM* (*PttPXY*). Etchells et al. (2015) observed that overexpressing *PttCLE41* or *PttPXY* in hybrid poplar resulted in taller trees with increased stem diameter due to higher cambial cell proliferation and xylem cell formation (Etchells et al., 2015). Zhu et al. (2020) observed an A-type peptide, PtrCLE20, produced by the secondary xylem moving into vascular cambium (Figure 2B) which suppresses its proliferation. Plants overexpressing *PtrCLE20* were shorter with decreased stem diameter due to significantly fewer vascular cambium and xylem cell layers (Zhu et al., 2020). Together, two poplar CLE peptides act in a complementary manner to retain an optimum number of dividing vascular cambium and differentiated xylem layers. However, it is not clear which stage of proliferative cell division each of the poplar CLEs regulate.

In addition to triggering proliferation of procambium/cambium and controlling its eventual differentiation, as discussed above, CLE41 also provides positional cues to control orientation of formative division. CLE41 diffuses from phloem to cambium (Figure 2B), thus potentially establishing a gradient and signals *via* PXY in the dividing cambial cells. This may alert them of the orientation of phloem and xylem tissues, thus assigning their plane of division, and signal to the daughter cell closest to the phloem to differentiate into a phloem cell. When Etchells et al. (2015) expressed *CLE41* ubiquitously, cell division orientations were disorganized and the arrangement of phloem, procambium and xylem was disarrayed (Etchells and Turner, 2010; De Smet and Beeckman, 2011; Etchells et al., 2015; Xu et al., 2021). In summary, like CLV3 in SAM, it is possible that CLE41 is secreted as a decision-making paracrine signal to proliferate procambium based on the current phloem differentiation status (stage 0—Figure 1B). In addition, during cambium differentiation into phloem and xylem, CLE41 could act as a polarizing signal to orient the cell division plane during stage 2 of cellular differentiation (Figure 1A).

In the meristematic zone of the root, xylem precursors in the protoxylem cell file position undergo periclinal divisions to increase the xylem file number of the metaxylem cell file positions. CLE9 is expressed in xylem precursors (particularly of the protoxylem cell file positions) and suppresses these divisions by binding BAM1, which is expressed widely across the vascular zone including xylem precursors (Figure 2I). BAM2 and -3 are also involved in this process. Thus, CLE9-BAM signaling restricts the xylem precursor cell proliferation, thereby preventing the formation of excessive number of differentiated metaxylem cell files (Qian et al., 2018). At the cellular level, CLE9-BAM1 signaling could be regulating any of the stages of proliferation—by suppressing the decision to proliferate or by regulating the cell cycle or any other cellular process (stages 0–2).

#### In stomatal lineage development

Meristemoid mother cells (MMC) are the stomatal lineage stem cells which give rise to the majority of the pavement cells (PC) in leaves by undergoing proliferative divisions and subsequent differentiation. An MMC first divides asymmetrically into a large stomata lineage ground cell (SLGC) and a smaller meristemoid cell, which in turn may undergo further rounds of asymmetric divisions forming more meristemoid cells. SLGC and meristemoid cells then differentiate into PCs and stomatal guard cells (GC), respectively (Lau and Bergmann, 2012). CLE9, expressed in MMCs, meristemoids and GCs, binds and signals *via* the RLK HAESA-LIKE 1 (HSL1) expressed in MMCs, meristemoids and PCs likely in an autocrine manner (Figure 2J). CLE9-HSL1 binding, which is enhanced by the SOMATIC EMBRYOGENESIS RECEPTOR KINASE 1 (SERK1) co-receptor, phosphorylates and destabilizes the TF SPEECHLESS (SPCH). SPCH is crucial for preserving MMC identity, and it also enables MMC to undergo subsequent asymmetric divisions that maintain the density of GCs and PCs in leaves (Qian et al., 2018). Thus, CLE9-HSL1 autocrine signaling in MMCs, by removing their own identity, unmarks themselves from being selected for formative divisions. Thereby, MMC differentiation is inhibited already at stage 1, when the cell to undergo division gets selected (Figure 1A).

#### In root nodulation

Nodules are specialized root organs that enable leguminous plants to enter symbiotic relationship with rhizobial bacteria. In response to rhizobial infection, some root cortical cells dedifferentiate, proliferate and finally form nodule primordia which host the bacteria. Nodulation is a costly process; hence plants exert a tight control over it through a process called autoregulation of nodulation (AON), which suppresses excessive nodulation (Ferguson et al., 2010; Reid et al., 2011). In *Medicago truncatula*, MtCLE12,-13 and -35 are expressed in the root in response to rhizobium and they mediate AON by signaling (by possibly binding) *via* the CLV1-like RLK SUPER NUMERIC NODULES (MtSUNN) expressed in the shoot. Thus, AON is likely the result of endocrine signaling from root to shoot (Imin et al., 2018; Lebedeva et al., 2020a; Mens et al., 2021). Similarly, in *Lotus japonicus*, root-derived arabinosylated LjCLE-RS1 and -2 are transported through xylem to the shoot where it binds and signals *via* CLV1-like HYPERNODULATION ABERRANT ROOT FORMATION 1 (LjHAR1), which is likely expressed in the leaf phloem (Krusell et al., 2002; Searle et al., 2003; Nontachaiyapoom et al., 2007; Okamoto et al., 2013). Observations on AON regulation by root-shoot CLE-RLK signaling in leguminous plants, demonstrated a novel role for CLE peptides as an endocrine signal (Okamoto et al., 2016). Moreover, the CRN and CLV2 orthologues in *M. truncatula*, MtCLV2 and MtCRN interact with MtSUNN; and mutation in *MtCRN* resulted in hypernodulation (Crook et al., 2016). Similarly, in *Lotus japonicus,* mutations in *LjCLV2* and in *RPK2* orthologue, *KLAVIER* (*LjKLV*) also resulted in hypernodulation. Moreover, LjKLV and LjHAR1 interact with each other (Miyazawa et al., 2010; Krusell et al., 2011). This indicates that nodulation is regulated by CLE signaling through CLV1-like RLKs and other receptors, such as CRN, CLV2, and KLV.

*MtCLE13* is already expressed in initial stages of nodule development. In the incipient and developing nodule primordium, *MtCLE13* is expressed strongly in the dividing cortical cells and mildly in pericycle cells around the regions of bacterial infection. But in the later young, round nodule stage, it is expressed throughout. However, *MtCLE12* is not expressed in the developing nodules, but in the later young, round nodule stage where, like *MtCLE13*, it is also expressed throughout the mature primordium (Mortier et al., 2010). It is likely that different CLE peptides produced by proliferating and differentiating cells during different stages of primordia development send paracrine AON activation signals in response (Figure 2C; Reid et al., 2011). Consistently, in response to AON activation, downstream hormones, TFs and miRNA (as observed in different leguminous plants) suppress nodule development (Suzaki et al., 2012; Takahara et al., 2013; Tsikou et al., 2018; Lebedeva et al., 2020b). Notably, in *Lotus japonicus*, this suppression was shown to occur after a few initial cortical cell divisions preventing the ensuing proliferative divisions, thus suppressing nodule primordium formation. Understanding which specific stage of cell division is being suppressed and the molecular mechanisms behind it will give us deeper insights into AON response signaling.

#### In nematode infection

Parasitic nematodes, such as root knot and cyst nematodes (RKN and CN, respectively) invade plant roots. The juvenile nematodes typically select a pericycle or procambial cell and inject several effector proteins including CLE peptide mimics into the cells through their stylet. The CLE mimics then reach the apoplast (Figure 2G) and manipulate the root vasculature development by inducing dedifferentiation and further proliferative divisions. The proliferated cells dissolve their cell wall and fuse to form a differentiated multinucleate feeding cell called a giant cell (in RKN) or syncytium (in CN), which serve as a nutrient source (Davis and Mitchum, 2005; de Almeida Engler and Gheysen, 2013; Frey and Favery, 2021).

In CN, A-type CLE mimics were identified in esophageal gland cell (Wang et al., 2005; Lu et al., 2009). Recently, Guo et al. (2015) identified also a B-type (TDIF)-mimic called *HsCLEB* (Figure 2G). They also observed high expression of *TDR* in the early syncytial stages in *Arabidopsis* after infection. TDR, CLV1, CLV2/CRN and RPK2 and WOX4 were shown to be crucial for syncytium growth and infection efficiency (Replogle et al., 2011; Guo et al., 2015, 2017). Furthermore, A-type HsCLE2 jointly with B-type HsCLEB could synergistically induce massive procambial cell proliferation through TDR (Whitford et al., 2008; Guo et al., 2017). Thus, it is likely that the CN TDIF mimic together with type-A CLE mimics hijacks the plant TDIF-TDR-WOX4 pathway to mediate syncytia formation during nematode infection (Guo et al., 2017). At the same time, in both galls and syncytia, there is an induced expression of procambial identity genes. This indicates that the nematodes maintain a pool of procambial stem cells by inducing proliferation possibly *via* the TDIF-TDR pathway for feeding cell formation (Yamaguchi et al., 2017). Learning how plant CLE-RLK signaling controls proliferation and at which stage(s) (Figure 1B) could support the generation of nematode resistant plant varieties in the future.

#### In organ primordium and organ development

Primordia and lateral organ development require controlled regulation of a series of processes: dedifferentiation, symmetric, asymmetric and proliferative divisions, and formative divisions and subsequent differentiation (Kwiatkowska, 2006; Torres-Martínez et al., 2019). CLE peptides have been observed to be functional in lateral root primordia (LRP) and lateral root (LR) development, and in flower and leaf primordia and correspondingly in flower and leaf outgrowths.

##### Lateral root primordium

*CLE26* is expressed at the phloem pole in the stele and increases LR density by controlling primary root (PR) length and LR numbers. It is hypothesized that CLE26 controls auxin transport, thus modulating the auxin distribution during LR initiation (Czyzewicz et al., 2015). Similarly, CLE41, CLE44 and a poplar-derived TDIF-like peptide have also been shown to promote LRP establishment and increase LR length by mediating auxin redistribution along the LR initiation sites and in emerging LRs (Yang et al., 2020).

LR development is also controlled by CLE peptides responding to environmental factors, such as nutrient availability and symbiosis with arbuscular mycorrhizal (AM) fungi and rhizobia (Araya et al., 2014; de Bang et al., 2017; Dong et al., 2019; Le Marquer et al., 2019). Araya et al. (2014) reported that CLE3-CLV1 signaling suppresses the number and length of emergent LRs, thus likely preventing the roots from expanding in low nutritional environment. *CLE3* was upregulated in N-deficient conditions particularly in pericycle cells of both PR and LR. Furthermore, CLE3 likely moves and signals *via* CLV1, localized in the phloem companion cells of both PR and LR (Paracrine signaling; Figure 2D). *clv1* showed a significant increase in LR length under N-deficient condition and the suppression of LR length by CLE3 was alleviated in *clv1*. However, it is not clear how the signaling operates at the cellular level (Figure 1) to influence root emergence past stage VII of LR development (Araya et al., 2014). Dong et al. (2019) reported similar results in S-deficient conditions. Upon S-starvation, the LR density decreased and *CLE2* and -*3* genes were upregulated. On the other hand, the rate of LR formation was enhanced upon S-starvation in *clv1* mutants. This shows that CLV1 suppresses LR formation in S-deficient condition (Dong et al., 2019). In summary, CLV1 and several CLE peptides are essential for lateral root formation in response to S and N in the environment.

##### Leaf primordium

Two CLE peptides with different but overlapping expression and activity in lateral organ primordia affect leaf shape. *CLE6* is expressed at the bases of young rosette leaves and of floral organs, whereas *CLE5* is expressed at the bases of young rosette leaves and of cauline leaves. CLE5 and 6 act downstream of leaf patterning factors in the leaf primordium to fine tune final leaf morphology. The mutants *cle5* and *cle5;cle6* show mild defects in leaf width, curvature and symmetry (DiGennaro et al., 2018). Details on CLE regulation of leaf morphology, such as how and where the leaf primordia-RLK(s) perceive CLE, and whether the cellular and tissue level effects they mediate are through regulation of proliferation or differentiation processes, remain open for investigation.

##### Flower primordium

Jones et al. (2021) reported temperature-oriented regulatory pathways directing reproductive development. *CRN* is expressed in early incipient primordium and throughout primordium formation, and was found to be required for primordial outgrowth and continuous flower production at lower temperatures (16°C–24°C). It functions in parallel with CLAVATA signaling in mediating these processes. Several CLE peptides, including CLV3, and the RLKs BAM1, -2 and CLV1 are involved in this CLAVATA signaling pathway although how they interact to regulate flower primordium outgrowth and complete flower formation is not yet characterized. At higher temperatures, these processes are controlled by the transcriptional repressor EARLY FLOWERING 3 (ELF3) that bypasses CLV2/CRN signaling (Jones et al., 2021).

### Regulation of non-developmental responses

Widely observed effects of CLE-RLK signaling pertain to proliferation or differentiation processes through cell division regulation. Accordingly, the ancestral role of CLE-RLK signaling, surmised by tracing back its effects in moss, is speculated to be regulation of meristematic cell division (Whitewoods et al., 2018; Whitewoods, 2021). However, this hypothesis stems from widely used experimental approaches in studying plants that relied on strong discernible phenotypes that predominantly involved proliferation or differentiation during meristem or primordium development. This may have led to a slightly skewed perspective, namely that CLE signaling predominantly affects plant development. However, plants continuously adapt to various external stimuli and respond to their changing needs. Therefore, refocusing on non-developmental transitory and conditional plant responses will broaden our understanding of CLE-RLK signaling. In the next paragraph, we discuss the roles of CLE-RLK signaling in dehydration response, phosphate homeostasis and temperature stress through processes besides proliferation or differentiation control.

#### In dehydration response and stomatal closure

CLE-RLK signaling facilitates dehydration stress response. After a few hours of dehydration stress, *CLE25* is upregulated in procambium of the vascular tissue in roots and it is transported through the vasculature to reach the leaves (Figures 2I,K), where CLE25 upregulates abscisic acid (ABA) biosynthesis enabling stomatal closure. In *cle25* mutants, ABA response to dehydration was abolished and the plants suffered heavy water loss. This CLE25-mediated stomatal response occurs through BAM1 and -3 in leaves (Figures 2I,K). Thus, CLE25 can convey water-deficiency signals from the root to the leaves acting as a long-range endocrine signal (Takahashi et al., 2018). Similar to *CLE25*, *CLE9* expressed directly in stomatal GCs also regulate stomatal closure (Figure 2K) during dehydration stress by activating *MITOGEN-ACTIVATED PROTEIN KINASE 3* and − *6* (*MPK3/6*) and *via* other downstream regulators, including ABA. *CLE9* overexpressing plants generally had smaller stomatal apertures and the leaves exhibited lower rate of water loss. Notably, unlike in the case of CLE25, BAMs are not the mediators of CLE9 signaling. Although the two CLEs might share the same downstream signaling components, the RLK that responds to CLE9 is not yet identified (Zhang et al., 2019). In brief, during dehydration stress, two disparate CLE peptides—CLE25 and -9—through disparate molecular events facilitate the same stomatal closure response. Thus, CLE peptides can also act as stress responders that signal specific cells or tissues to enable counteracting responses that does not involve cellular proliferation or differentiation regulation.

#### During AM fungal symbiosis

Plants enter symbiotic relationship with AM for phosphate acquisition, and this symbiosis is maintained through several regulatory signals including CLE peptides (Besserer et al., 2006; Genre et al., 2013; Kobae et al., 2018; Le Marquer et al., 2019; Müller et al., 2019). Strigolactone produced by the plant promotes fungal symbiosis (Akiyama et al., 2005). The phosphorous status of the plant and also the level of fungal colonization itself positively or negatively regulate the fungal symbiotic development (Menge et al., 1978; Breuillin et al., 2010). In *M. truncatula*, *MtCLE53* and *MtCLE33* are upregulated in response to fungal colonization and excess phosphate acquisition. Both genes were expressed in vascular tissue and *MtCLE53* was particularly upregulated near fungal colonies (Figure 2E). These peptides reduce the fungal colonization by downregulating genes involved in strigolactone biosynthesis and secretion. Overexpression of *MtCLE53* and − *33* resulted in significant reduction in the number of fungal entry sites together with increased expression of the RLK *MtSUNN*. The working model is that, as the AM fungal colonization rises, levels of MtCLE53 gradually increase in response. Subsequently, MtCLE53-MtSUNN signals to further upregulate *MtSUNN* expression (positive feedback) and suppress strigolactone levels, thus reducing the rate of colonization (negative feedback; Müller et al., 2019).

CLE genes have been identified also in some species of AM fungi, which enable them to manipulate plant processes for symbiosis. The CLE peptides *RiCLE1 and GrCLE1*from *Rhizophagus irregularis* and *Gigaspora rosea* are expressed at higher levels during the mycorrhizal state of the plant (Figure 2E). *Medicago truncatula* roots pre-treated with the peptide and further exposed to the spores showed higher number of fungal entry points with longer colonization sites (Le Marquer et al., 2019). To recapitulate, these root-acting CLEs produced during AM symbiosis respond transiently to the nutritional status of the plants and mediate phosphate homeostasis by directly regulating the fungal colonization. This exemplifies a CLE-RLK-mediated non-developmental effect.

#### In pollen tube viability

CLE-RLK signaling also mediates adaptational response to high-temperature stress. *CLE45*, expressed in stigma, expands its expression domain to the pollen tube transmitting tract after a shift toward higher temperature. CLE45 then acts as a paracrine signal and binds to the RLK STERILITY-REGULATING KINASE MEMBER1 (SKM1) expressed in pollen. CLE45 signaling *via* SKM1 and SKM2, which are both expressed in pollen, have been shown to prolong pollen tube viability at high temperatures by maintaining its mitochondrial activity until it reaches the ovules (Figure 2L). This is essential for pollen-embryo sac interactions and loss of CLE45 or the RLKs resulted in aberrant seed production (Endo et al., 2013). Thus, this heat-inducible CLE45-SKM1/2 system exemplifies CLE-RLK signaling in mediating a non-developmental response to abiotic stress.

### Conclusion and remarks: Temporal and spatial dimensions

1. The temporal dimension: Has it been seconds, minutes, hours, or days?

*Cellular effects:* The temporal dimension illustrates the time elapsed from the perception of CLE signal by the RLK—seconds to weeks, which correlates with the extent of the CLE effect across plant body organization—from molecular to organ or organism level. The most studied effects of CLE signaling are the regulation of proliferation and differentiation at tissue and organ level. However, detailed studies into the molecular mechanisms behind CLE mediating cell level changes might provide unique insights on proliferation and differentiation processes.

*Non-developmental effects*: Current research approaches that involve tracing the signaling events resulting in strong developmental phenotypes, very often, lead to uncovering defects in cell division regulation, hence limiting our understanding of the full range of CLE-RLK signaling effects. Signaling events that produce non-developmental effects which occur specifically during a narrow time window or after an external stimulus remain underestimated due to lack of a strong observable phenotypes. Therefore, exposing the plants to diverse conditions and analyzing their transcriptome and following the CLE peptide expression patterns would provide us with an extensive array of its molecular to organ level effects. So far, there are a few studies, as discussed in this review, that show CLE-RLK mediated non-developmental responses. Further studies in this respect across different clades (lycophytes, ferns and gymnosperms, including bryophytes) will provide a greater prospect of defining the ancestral role.

2. The spatial dimension: Who initiates the CLE signal, who receives it and how?

Understanding the spatial aspect of a signaling process is essential as it enables us to delineate the cells, tissue or organs that are in direct communication. For example, induction of stem cell proliferation in a given tissue could be a consequence of CLE peptide produced by the neighboring tissue (paracrine signal) where rapid differentiation takes place.

On the other hand, how the CLE signal reaches the destination is an essential part of the signaling process. The overall analysis of CLE signaling range show that auto- and paracrine CLE signaling is more common than endocrine. It is likely that establishing long-distance communication is challenging as it depends on several factors such as (1) controlling the expression level and concentration of the peptide which further depends on its stability (half-time), dilution during transport through xylem, duration, consistency and level of expression of the peptide (2) post-translational modification of the peptide (3) access to the transport system which includes expression pattern of both the peptide and RLKs, as they should be expressed in the vicinity of vascular tissue: CLE peptide for its extensive transport and RLK for conveniently binding the transported peptide molecules (4) conditions in the destination cells or tissue where further dilution or concentration of the peptide molecules can take place (Müller and Schier, 2011; Notaguchi and Okamoto, 2015; Okamoto et al., 2015, 2016). However, the factors that restrict or expand the CLE signaling range remain so far unknown.

## Promiscuity in CLE-RLK interaction

An important attribute of the CLE-RLK interaction is promiscuity. Promiscuity is a broad term used in two main contexts (Copley, 2015):

1. “Broader specificity”—This is when a protein can bind multiple ligands or interact with different proteins to mediate different effects. For example, “hub” proteins that contain disordered regions, which offer them conformational plasticity to serve as centers of signaling networks (Tsai et al., 2009; Schreiber and Keating, 2011).
2. “Physiologically irrelevant interactions”—This is when a protein interacts with a substrate in an artificial test scenario, which will not occur in the natural system. For example, a group of enzymes that are expressed as a part of an operon and function together will perform a particular process in the natural system. But one isolated enzyme, in a new environment, might perform an entirely new function owing to its active binding site that could bind another substrate (Copley, 2015).

Promiscuous interactions among proteins in a given system could be influenced by: (a) Sequence and/or structural similarity among the possible binding partners, which decides the binding affinity to a specific interacting partner compared to other proteins (Schreiber and Keating, 2011; Copley, 2015), (b) Co-evolution of the binding interfaces of the interacting proteins, which promotes binding specificity through positive or negative selection of binding residues (Moyle et al., 1994; Yosef et al., 2009; Schreiber and Keating, 2011), (c) Post-translational modifications that offer interaction specificity (Tsai et al., 2009; Schreiber and Keating, 2011), (d) Concentration of the interacting protein in the local environment. High affinity binding occurs in the nM to μM range. If the concentration of other molecules or proteins is in excess amount, it can lead to non-specific interactions (Schreiber and Keating, 2011).

### Examples of promiscuity in CLE signaling

Promiscuity has been observed among CLEs and RLKs during their interactions—be it natural or forced, such as in experimental simulations. Here, we have illustrated some of the common observations with examples.

– *Several CLE peptides (from the same or different species) causing the same effect*—Examples: Exogenous application of CLE25, CLV3, CLE46, and TDIF upregulate *NCED3* expression in leaves (Takahashi et al., 2018). Exogenous application of AtCLV3 and overexpression of *MpCLE2* result in the same effect—lateral expansion of the apical notch—in *Marchantia* (Hirakawa et al., 2020). Although these observations suggest that the same RLK perceives several CLEs, their interactions are physiologically irrelevant. This is because these interactions are likely the result of very high local peptide concentrations perceived in non-specific tissue layers leading to non-specific promiscuous interactions.
– *A peptide binding to different RLKs with strong affinities*—Examples: Through binding experiments, CLE9 has been shown to bind to both BAM1 and HSL1 with high affinities; Similarly, CLE45 can bind to both BAM3 and SKM1 (Shinohara et al., 2012; Endo et al., 2013; Hazak et al., 2017; Qian et al., 2018). This could be the result of RLKs and CLEs not establishing strict negative or positive selection during co-evolution. However, it is to be noted that co-receptor binding has been shown to offer higher binding affinities in the case of CLE9-HSL1 interaction (Qian et al., 2018) and importantly RLKs and the CLEs might undergo post-translational modifications that could confer binding specificity.
– *One RLK replacing the other spatially and functionally*—Examples: In *clv1* mutant, *BAM1* is upregulated and its expression shifts from PZ to OC and at least partially suppresses IFM expansion by binding CLV3 and signaling in the CLV pathway (DeYoung et al., 2006; Nimchuk et al., 2015; Schlegel et al., 2021). Furthermore, all meristem defects of *clv3* mutants were fully rescued by *CLE40* when expressed from the *CLV3* promoter (Hobe et al., 2003). These observed functional compensation mechanisms are the results of broader specificity of CLV3 and CLE40 in binding to BAM1 and CLV1—two closely related RLKs of the same subfamily, but *BAM1* and *CLV1* are naturally expressed in different zones, and they will therefore, *in vivo*, likely interact with only one of these CLEs, respectively.
– *Multiple CLEs signaling through same RLKs*—Example: Several redundant CLEs have been shown to compensate for CLV3 loss-of-function in suppressing IFM expansion. These redundant genes have been implicated to be consistently expressed in the inflorescence apices and not simply upregulated as a consequence to CLV3 loss, i.e., in *clv3*. CLV3 and these non-paralogous CLEs signal through (likely by binding to) CLV1 and BAM family of RLKs (Rodriguez-Leal et al., 2019). This observation suggests broader binding specificity of RLKs to several CLEs in a natural system, *in vivo*.

In summary, some of these observations exemplify promiscuous CLE-RLK interactions, albeit with lower binding affinity or at physiologically irrelevant concentration or at extraneous expression domains and time. However, other promiscuous interactions are on the account of their high structural similarity among peptides or RLKs of the same subfamilies.

### Regulatory aspects of CLE-RLK interaction that promote signaling specificity

**Figure 3:**
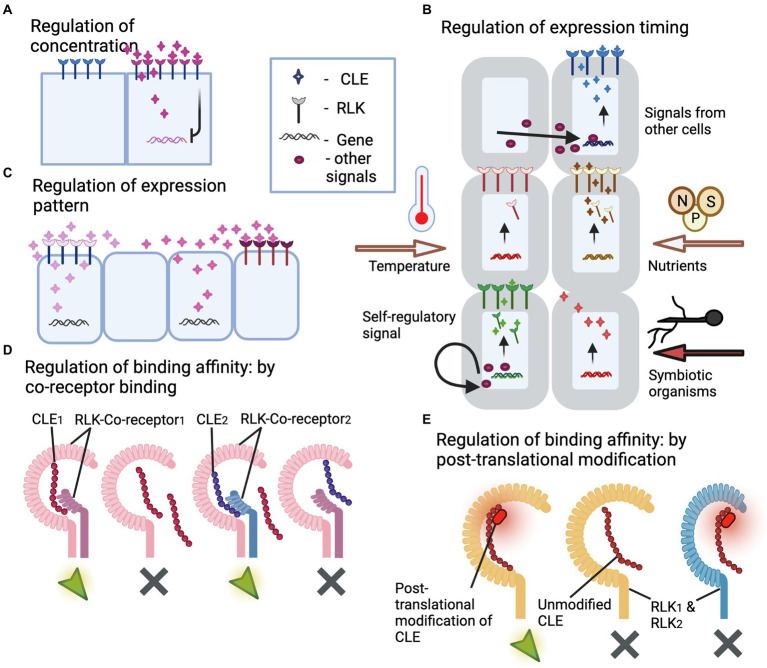
Illustrations of regulatory mechanisms that prevent cross-talk between CLE peptides and RLKs. **(A)** Regulation of CLE peptide concentration. The illustration depicts CLE peptide regulating its own synthesis through negative feedback loop, thus containing itself from excessive expression and spreading leading to cross-talk with other RLKs. **(B)** Regulation of the timing of CLE and/or RLK expression. The illustration depicts CLE and RLK expression controlled by external stimuli like temperature, nutrient availability, colonization by a symbiotic organism, or after certain regulatory signals produced by the same or other cells. Thus, the temporal control of expression pattern prevents cross-talk and competitive binding. **(C)** Regulation of the spatial expression pattern of CLEs and RLKs. This illustration depicts spatially separated expression of two similar CLEs avoiding cross-talk with the other RLK. Thus, the spatial control of expression pattern prevents cross-talk and competitive binding. **(D)** Regulation of CLE-RLK binding affinity through co-receptor binding. The illustration depicts co-receptors offering high binding affinity and thus high specificity to CLE-RLK interaction leading to activation of downstream signaling with no cross-talk. CLEs exhibit high affinity to the RLK that is bound with the corresponding co-receptor (co-receptor1 for CLE_1_; co-receptor2 for CLE_2_), whereas they exhibit low binding affinity to the co-receptor unbound RLK or to the RLK bound to a different co-receptor (co-receptor1 for CLE_2_). **(E)** Regulation of CLE binding affinity through post-translational modifications. The illustration depicts post-translational modification offering high binding affinity and thus high specificity to CLE-RLK interaction leading to activation of downstream signaling with no cross-talk. The CLE peptide with post-translational modification binds to the corresponding RLK (RLK_1_) with higher affinity compared to its unmodified counterpart and thus avoids cross-talk with the non-specific RLK_2_. The green arrows and gray crosses in **(D,E)** represent activated and non-activated downstream signaling.

Why do CLEs and RLKs exhibit promiscuous interactions? It could be because of residual interactive motifs that are relics of ancestral functions. In an environment of highly similar proteins, avoiding cross-interaction through “perfect” binding site might be impossible and might be a costly process. Importantly, natural selection moderates the traits that lower the fitness of the organism. Hence, if the promiscuity does not disrupt the CLE-RLK downstream signaling due to cross-talk or antagonism, there will be no selective pressure against it (Copley, 2015). To that end, there are several regulatory aspects to CLE-RLK interaction that minimizes their cross-talk and fosters specificity in downstream signaling. In this section, we have described such observed regulatory aspects that likely prevent detrimental effects due to cross-talk, thereby bypassing the selective pressure toward binding specificity (Figure 3).

#### Regulating the concentration of CLEs

Several CLEs and RLKs function in proximity from each other. If the concentration of a secreted CLE increases over a threshold, there could be non-specific interactions with neighboring RLKs resulting in detrimental effects, which might lead to negative selection pressure necessitating co-evolution of binding specificity. There are established feedback loops and other repressive controls in CLE-RLK signaling networks, which maintain stable CLE concentration. For example: the CLV3-CLV1-WUS negative feedback loop maintains stable CLV3 concentration in *Arabidopsis* SAM. Similarly, SlCLV3 negative feedback represses its own expression (*SlCLV3*) and of its closest paralogue *SlCLE9* in tomato SAM (Yadav et al., 2011; Rodriguez-Leal et al., 2019).

#### Regulating the timing of CLE/RLK expression

Controlling when each CLE/RLK is expressed limits the possibility of cross-talk and competition with other CLEs/RLKs. Several external stimuli (nutrients, temperature, symbiosis, etc.) and internal developmental signals trigger CLE/RLK expression, thus controlling their expression timing—Table 1. Example: *CLE25* is upregulated in root vasculature only during dehydration stress.

#### Regulating the CLE/RLK spatial expression pattern

Similar to the timing of expression, the spatial expression pattern also limits non-specific interactions. CLE/RLK expression in cells/tissue is directly controlled by TFs, which in turn are regulated by the developmental and physiological needs. Example: WUS restricts the *CLE40* expression zone in the PZ in response to the proliferation/differentiation status of the SAM (Schlegel et al., 2021).

#### Co-receptor binding

Co-receptors regulate the binding specificity of RLKs toward peptides possibly by binding to and conferring a conformational change of the RLKs, thereby making it more or less specific for certain CLEs (Olsson et al., 2019). To that end, SERK1 increased the binding affinity between CLE9/10 and HSL1. But another RLK, BAM1, while binding to the same CLE9/10 peptide, did not recruit SERKs; hence, BAM1 functions either without any co-receptor recruitment or recruits some other co-receptor that subsequently increases its affinity to CLE9/10. Thus, co-receptors confer differential binding affinities for RLKs to mediate specific CLE-RLK interaction, thereby preventing RLK cross-talk with other CLEs.

#### Post-translational modifications of CLEs

Post-translational modifications alter the conformational properties of the proteins and change their binding specificities (Tsai et al., 2009; Schreiber and Keating, 2011). CLE peptides have been shown to undergo different post-translational modifications, some of which strengthen their binding affinity with the RLKs (Stührwohldt et al., 2020). Example: Tri-arabinosylated CLV3 binds CLV1 receptor with higher affinity than unmodified CLV3 *in vitro* (Ohyama et al., 2009). Such post-translational modifications could also possibly result in specific binding of CLEs to their respective RLKs, thus effectively preventing cross-talk with other RLKS.

## Conclusion and remarks: CLE-RLK interaction and signaling

In conclusion, the CLE expression level, timing and spatial pattern, together with co-receptor binding limits CLE-RLK interactions to a specific region, time and type; thereby non-specific interactions or cross-talks are prevented *in vivo*. This has largely eliminated the evolutionary pressure to improve binding specificity between CLEs and RLKs, which has resulted in a general promiscuity in CLE-RLK interaction.

On the other hand, promiscuity or broader binding specificity among the signaling ligands and their receptors might offer an evolutionary advantage. It was shown that, in bone morphogenetic pathway, “combinations of different ligands” act more efficiently when signaling to a group of cells of different types expressing different receptors, compared to “one-to-one” ligand-receptor specific binding. Varied concentrations and combination of ligands were able to activate or deactivate different receptors and their downstream signaling (Su et al., 2022). Similar to their observations, it is possible that several CLEs together mediate such a complex combinatorial regulation during IFM development when several CLEs are expressed, and possibly during other processes too. In that case, mutagenesis and over expression analyses might be non-viable for studying CLE-RLK interaction and signaling. Synergistic effects of CLE peptides have been reported before (Whitford et al., 2008). However, attempting to reproduce *in vivo* levels and combinations is pivotal in understanding the nuances in promiscuous CLE-RLK signaling.

Recently several strides have been made toward understanding co-receptors regulating CLE-RLK signaling (Olsson et al., 2019; Gou and Li, 2020). There are still several avenues of CLE-RLK interactions that remain unexplored:

- Post-translational modifications in CLEs and RLKs: What are the different ways in which both RLKs and CLEs are modified? How are these modifications regulated? How do they affect CLE-RLK interaction?
- RLK level in the cell/tissue: How are RLK turnover, inactivation/activation and expression level controlled? And how do they affect CLE signaling?
- Can variations in concentration and combination of multiple CLEs expressed in a region activate/deactivate the RLKs of that region?
- How is the signaling range (auto−/para−/endocrine) controlled? Can it be achieved solely by regulating the CLE-RLK expression pattern and levels?
- What are the downstream signaling components of CLE-RLK and, by extension, the downstream effects that are conserved across clades? Understanding the differences in closely related non-angiosperm vascular plants will already be insightful.

## Author contributions

MN and RS conceived the idea. MN wrote the manuscript and made the figures. All authors contributed to the article and approved the submitted version.

## Funding

MN was supported by the DFG through a grant within the CRC1208 to RS.

## Conflict of interest

The authors declare that the research was conducted in the absence of any commercial or financial relationships that could be construed as a potential conflict of interest.

## Publisher’s note

All claims expressed in this article are solely those of the authors and do not necessarily represent those of their affiliated organizations, or those of the publisher, the editors and the reviewers. Any product that may be evaluated in this article, or claim that may be made by its manufacturer, is not guaranteed or endorsed by the publisher.
